# Adaptation of a fair individualized polysocial risk score for hospitalization risk prediction

**DOI:** 10.1093/jamiaopen/ooag161

**Published:** 2026-08-24

**Authors:** Qinglin Gou, Yu Hu, Xing He, Megan E Gregory, Jennifer H LeLaurin, Ramzi G Salloum, Jiang Bian, Jingchuan Guo, Yu Huang

**Affiliations:** Department of Health Outcomes and Biomedical Informatics, College of Medicine, University of Florida, Gainesville, FL 32610, United States; Department of Biostatistics and Health Data Science, School of Medicine, Indiana University, Indianapolis, IN 46202, United States; Regenstrief Institute, Indianapolis, IN 46202, United States; Department of Biostatistics and Health Data Science, School of Medicine, Indiana University, Indianapolis, IN 46202, United States; Regenstrief Institute, Indianapolis, IN 46202, United States; Indiana University Melvin and Bren Simon Comprehensive Cancer Center, Indianapolis, IN 46202, United States; Department of Health Outcomes and Biomedical Informatics, College of Medicine, University of Florida, Gainesville, FL 32610, United States; Department of Health Outcomes and Biomedical Informatics, College of Medicine, University of Florida, Gainesville, FL 32610, United States; Department of Health Outcomes and Biomedical Informatics, College of Medicine, University of Florida, Gainesville, FL 32610, United States; Department of Biostatistics and Health Data Science, School of Medicine, Indiana University, Indianapolis, IN 46202, United States; Regenstrief Institute, Indianapolis, IN 46202, United States; Indiana University Melvin and Bren Simon Comprehensive Cancer Center, Indianapolis, IN 46202, United States; Regenstrief Institute, Indianapolis, IN 46202, United States; Department of Pharmacy Practice, College of Pharmacy, Purdue University, Indianapolis, IN 46202, United States; Department of Biostatistics and Health Data Science, School of Medicine, Indiana University, Indianapolis, IN 46202, United States; Regenstrief Institute, Indianapolis, IN 46202, United States; Indiana University Melvin and Bren Simon Comprehensive Cancer Center, Indianapolis, IN 46202, United States

**Keywords:** machine learning, social determinants of health, predictive model, fairness

## Abstract

**Objectives:**

We adapted the individualized polysocial risk score (iPsRS), a machine learning model originally developed for patients with type 2 diabetes, to evaluate its generalizability in predicting 1-year hospitalization risk in a disease-agnostic adult cohort, with attention to fairness and explainability.

**Materials and Methods:**

The study utilized de-identified electronic health record data from a retrospective cohort of 17 857 adult patients at the University of Florida Health. The original iPsRS framework was reimplemented using XGBoost and Logistic Regression models. These models were trained and fine-tuned using 13 individual-level social determinants of health (SDoH) predictors to predict all-cause hospitalization within 1 year. Models were fine-tuned at 5 levels (0%, 10%, 20%, 50%, and 70%) with 3 sampling strategies (none, oversampling, and undersampling). Model performance was primarily evaluated using the AUROC. Interpretability was assessed through SHapley Additive exPlanations and a causal structure learning algorithm, while fairness was analyzed by examining false negative rate disparities across age, sex, and racial/ethnic subgroups.

**Results:**

After fine-tuning, the adapted iPsRS demonstrated moderate predictive performance, achieving an AUROC of up to 0.671 with XGBoost and Normal sampling. SHapley Additive exPlanations analysis on the evaluation split showed age, food insecurity, marital status, and financial constraints emerged as consistently influential predictors. The causal structure analysis corroborated predictive findings, identifying age, race, and employment as proximal factors associated with hospitalization.

**Conclusion:**

The iPsRS was successfully adapted beyond diabetes, maintaining moderate predictive performance and meaningful risk stratification. Incorporating individual-level SDoH enables equity-aware prediction, supporting broader use of iPsRS in clinical care.

## Introduction

The social determinants of health (SDoH)—including education, employment, financial stability, housing, and behaviors such as smoking or drug use—are now well-established as critical contributors to individual and population health outcomes.^1–3^ It is estimated that up to 50% of health outcomes can be attributed to non-medical social and environmental factors.^4^ Specific domains such as financial stress, housing insecurity, and substance use are strongly linked to preventable hospitalizations and health disparities across populations.^5–7^ As such, efforts to integrate structured SDoH screening tools into clinical workflows have gained widespread momentum in recent years, supported by clinical guidelines and professional advocacy.^8–10^ The Centers for Medicare & Medicaid Services have mandated inpatient screening of selected SDoH needs starting in 2024.^11^ Despite growing national attention, wide-scale screening of SDoH has not yet become standard practice.^12^^,^^13^ One key reason for the low rate of SDoH screening is limited resources; most healthcare systems operate under resource-constrained conditions and lack the necessary infrastructure and incentives to implement adequate social needs screening.^14^^,^^15^ Additionally, although validated SDoH screening tools are available, these tools are not automated, making them difficult to integrate efficiently into clinical workflows.^16^^,^^17^ A computational framework to stratify patients at high risk of unmet social needs is therefore essential for enabling automated, scalable SDoH screening.

Machine learning (ML) has shown promise in translating complex SDoH screening into actionable risk stratification. Various ML approaches, such as logistic regression, random forests, and XGBoost have predicted outcomes such as quality of life, cardiovascular disease, and heart failure using structured SDoH features.^18–20^ However, generalizability remains a critical challenge. Zhao et al. emphasized that ML models trained on SDoH data often underperform when applied to new populations due to regional, demographic, and systemic variations in how SDoH are expressed and recorded.^21^ For instance, performance disparities are observed when models developed in high-income or urban settings are transferred to low-resource or rural populations, especially when social or environmental variables are not uniformly represented. Clark et al. found that while ML models predicted self-rated health with good discrimination (AUC ∼0.81), predictors varied significantly by age, sex, and race/ethnicity—highlighting how subgroup heterogeneity affects feature importance and accuracy.^22^

Against this backdrop, our team previously developed the individualized polysocial risk score (iPsRS) to predict 1-year hospitalization in patients with type 2 diabetes (T2D), incorporating both individual-level (eg, housing stability, insurance status) and contextual-level (eg, neighborhood deprivation) SDoH.^23^ Using XGBoost and SHapley Additive exPlanations (SHAP) for interpretability, the model demonstrated strong predictive performance in the T2D cohort, highlighting its potential to identify patients at elevated social risk for adverse outcomes.^24^ However, a critical limitation remains: the generalizability of iPsRS beyond disease-specific cohorts has not yet been evaluated. This gap is especially concerning because, as models incorporating SDoH gain traction in clinical risk prediction, ensuring their performance and fairness across diverse, externally validated populations becomes vital. Recent work emphasizes that models trained on narrow or disease-specific cohorts often underperform when externally validated in broader, demographically heterogeneous populations.^25^ For instance, when a deep reinforcement learning framework was externally validated across 3 independent hospitals, significant performance and fairness improvements were observed compared to static models, revealing the need for site-specific bias mitigation. Furthermore, differences in electronic health record **(**EHR) structures, regional coding standards, and SDoH measurement practices can hinder model transferability, necessitating robust external validation procedures.^26^ Even when models achieve good performance in internal settings, subgroup misclassification—particularly for minority racial or low-income groups—can remain unaddressed. For example, Thompson et al. found that an NLP-based opioid misuse classifier exhibited a false negative rate (FNR) of 32% in Black patients vs 17% in White patients, a disparity that required post-hoc recalibration to correct.^27^ Complementary work by Wang et al. also highlights the risk of racial bias in predictive models for chronic disease mortality, demonstrating that failing to account for underlying SDoH differences—such as insurance status and education—can misrepresent fairness performance across cohorts.^28^ Thus, external validation and fairness auditing remain critical steps before these models are applied in real-world clinical or policy settings.

To address this, we adapted the T2D-specific iPsRS for a general adult cohort using 13 structured SDoH features (age, sex, race/ethnicity, insurance, education, employment, financial constraints, housing, food insecurity, marital status, and substance use). We evaluated discrimination and calibration, interpretability via SHAP and causal structure learning, and subgroup fairness using FNR disparities. This work builds upon growing research emphasizing that SDoH-informed models must be externally validated across diverse populations to ensure robustness and generalizability.^29–31^ Meanwhile, algorithmic fairness has emerged as a critical area of concern, with recent efforts demonstrating methods to mitigate bias and improve equitable outcomes for underserved populations.^32^^,^^33^ By extending iPsRS from a disease-specific context to a general population, we seek to determine whether social risk prediction can be generalized beyond T2D, ultimately supporting more inclusive and effective population health management.

## Methods

### Data source and study cohort

This study used de-identified patient data from a retrospective cohort approved under IRB202201652. In contrast to the original iPsRS development cohort, which was limited to patients with T2D and derived individual-level SDoH from clinical notes using an NLP pipeline, the adaptation cohort in this study was independently constructed as a general adult primary care population based on self-reported SDoH data. The 2 cohorts were assembled under different IRB protocols and through separate data extraction pipelines and are therefore analytically distinct and not directly linked.

The dataset included 17 857 adult patients (≥18 years) receiving care within University of Florida (UF) Health primary care clinics. Patients were identified through the epic EHR via the UF Health Integrated Data Repository, with inclusion restricted to those who had documented baseline self-reported SDoH information collected as part of routine clinical screening. The cohort was drawn from UF Health primary care sites across both the Gainesville and Jacksonville campuses and included encounters spanning January 1, 2018-June 30, 2022.

The primary outcome was defined as all-cause hospitalization within 1 year, identified based on documented inpatient encounters in the EHR. The index date was determined as the first encounter during which baseline SDoH documentation was available. A total of 747 patients (4.2%) experienced at least 1 hospitalization during the follow-up period.

The final set of predictor variables included 13 covariates, grouped into 2 categories: (1) demographic and (2) individual-level SDoH. Demographic covariates included age, sex, and race/ethnicity. The SDoH variables were primarily derived from structured data sources. Employment status was the only variable supplemented using information extracted from unstructured clinical notes through a validated rule-based natural language processing (NLP) pipeline to capture employment-related information not available in structured fields. This pipeline employed a conservative, domain-informed keyword strategy as an initial screening step to reduce false-positive identification, rather than broad semantic matching.^34^ Individual-level SDoH variables included insurance coverage, education level, employment status, financial constraints, housing stability, food insecurity, marital status, and health-related behaviors, including smoking, alcohol use, and drug use. All variables were coded as categorical or ordinal indicators using harmonized value sets. For example, housing status was categorized as “homeless/shelter,” “stable housing,” or “other,” and financial constraint was classified as “yes,” “no,” or “other.” Missing or insufficiently documented responses were retained as “unknown” or “other” categories rather than statistically imputed, reflecting real-world EHR documentation practices and preserving information about documentation status. A complete listing of all covariates, coding schemes, and variable sources is provided in **Table S1**.

### iPsRS model overview

The individualized iPsRS was previously developed using a cohort of patients with T2D from the UF Health system.^23^ The original model incorporated both individual-level and contextual-level SDoH and was trained using 2 supervised ML algorithms: extreme gradient boosting (XGBoost) and ridge regression. Of the 2, XGBoost achieved superior performance and was selected as the final iPsRS source model.

**Figure 1.**
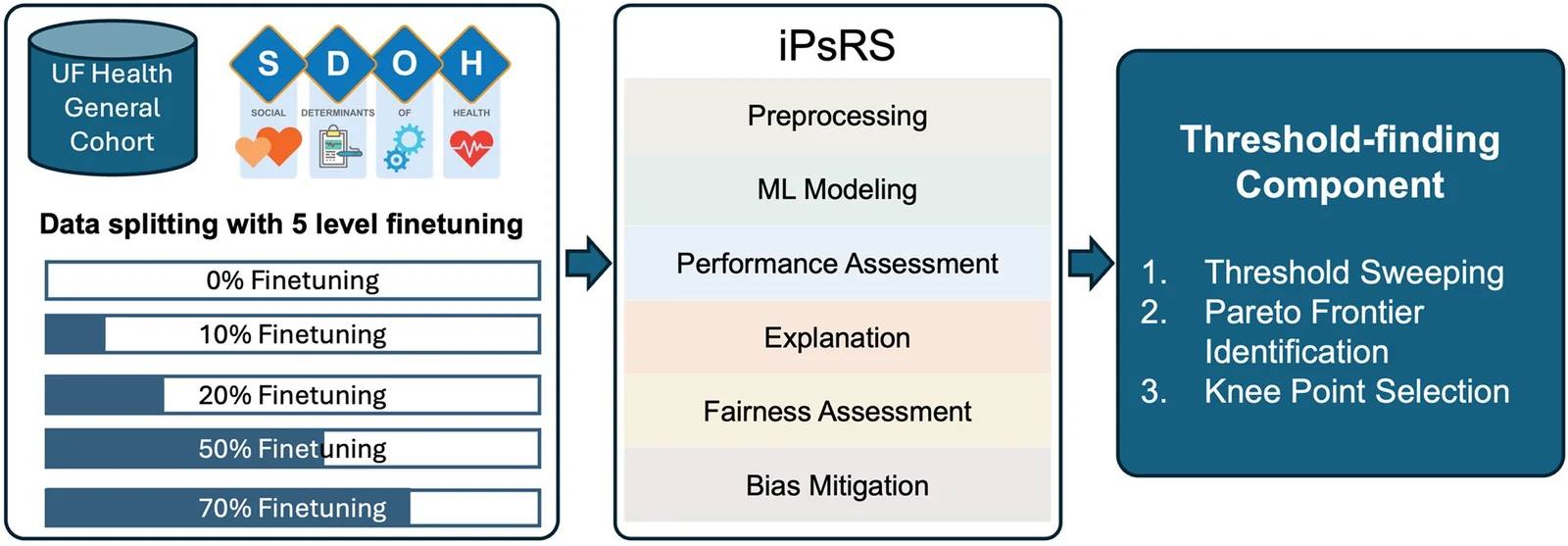
Framework for iPsRS adaptation and evaluation. The framework illustrates model fine-tuning and evaluation using UF Health SDoH data, including performance, fairness, explainability, and threshold selection components.

In this study, we aimed to evaluate the generalizability of the iPsRS framework by applying it to a general adult patient population that only included individual-level SDoH features. To do this, we implemented the model architecture using the original iPsRS configuration and transferred it to apply to a general adult patient population. This design allowed us to explore the adaptability of the original model concept across a different population and limited feature set, while preserving the same prediction task of 1-year all-cause hospitalization. We also extended the original framework by introducing a threshold-finding component to support real-world deployment. This addition enables identification of an optimal predictive threshold. An overview of the adapted iPsRS framework and evaluation pipeline is shown in Figure 1.

### Threshold-finding component

To determine the optimal classification threshold for model deployment, we employed a structured 3-stage approach, consisting of (1) threshold sweeping, (2) Pareto frontier identification, and (3) knee point selection.^35–37^ This approach explicitly balances multiple performance objectives and allows for post-hoc selection of the decision threshold.

#### Stage 1—Threshold sweeping

For the final selected model configuration, predicted probabilities were converted to binary predictions using thresholds from 0.01 to 0.99 (increment = 0.01). At each threshold, we calculated precision, recall (sensitivity), specificity, and F1 score. Area under the receiver operating characteristic curve (AUROC) was also recorded, though it remained constant across thresholds.

#### Stage 2—Pareto frontier identification

We defined a threshold as *Pareto-optimal* if no other threshold achieved equal or higher performance across all selected metrics with strictly higher performance in at least one.^38^ Formally, threshold $x_{a}$ dominates threshold $x_{b}$ if:


$$\forall m,f_{m}(x_{a})\geq f_{m}(x_{b})\;a\mathrm{nd}\;\exists \;m:f_{m}(x_{a})\;>\;f_{m}(x_{b})$$

where $f_{m}\;$denotes the value for metric m in {precision, recall, specificity, F1 score}. Non-dominated thresholds were assigned to the Pareto set (level 0), representing the set of equally optimal tradeoff solutions. Specificity was included among the Pareto objectives to constrain trivial high-recall solutions that would classify nearly all patients as high risk, rather than as a primary optimization target.

#### Stage 3—Knee point selection

From the Pareto set, we applied the minimum Manhattan distance approach to find a knee-point, representing the most balanced solution.^39^ This procedure can be run in 2 modes. In the normalized mode, each metric was scaled to the [0,1] range, and the utopia point was defined as (1,1,…,1). In the non-normalized mode, raw metric values were used and the utopia point was defined by the maximum value of each metric within the Pareto set. In both cases, the Manhattan distance to the utopia point was computed as:


$$D_{\mathrm{Manhattan}}(x)=\Sigma_{m}\;|1-f_{m}(x)|$$

The knee point was defined as the threshold with the smallest Manhattan distance, representing the most balanced tradeoff among precision, recall (sensitivity), specificity, and F1 score. Specificity was retained as an operational guardrail rather than as the primary clinical objective, to avoid trivial high-recall thresholds that would classify nearly all patients as high risk. Cohort size was considered only at the knee-point selection stage as an operational feasibility measure, not as a criterion for defining Pareto dominance, and was used to avoid extreme thresholds that would result in either an impractically large high-risk population or an insufficient number of identified cases under class imbalance.

### Experimental design

To evaluate the transportability of the original iPsRS model, we conducted a series of fine-tuning experiments using a general adult patient dataset distinct from the original T2D cohort. The original iPsRS model weights were used as the initialization point, and fine-tuning was performed on progressively larger subsets of the general dataset to simulate varying levels of model adaptation. Five fine-tuning configurations were tested: 0%, 10%, 20%, 50%, and 70%, representing the proportion of general cohort data used to update the model parameters. The 0% setting involved direct application of the pre-trained iPsRS model without any local retraining, while higher percentages reflected increasing levels of model exposure to site-specific data. A held-out portion of the dataset was reserved for evaluation in each configuration.

The general cohort dataset (*n *= 17 857) was randomly split into stratified training (70%, *n *= 12 502) and held-out test (30%, *n *= 5 355) sets, with the test set fixed across all experiments. Given the substantial class imbalance in the outcome (only 4.2% of patients experienced hospitalization within 1 year), we applied 3 sampling strategies during model training to assess their impact on performance and fairness. These resampling strategies were included as controlled experimental factors, rather than as methods assumed to improve model performance. These included: (1) no resampling (Normal) with 12 502 training samples, (2) random oversampling (ROS) of the minority class with 23 954 training samples, and (3) random undersampling (RUS) of the majority class with 1 050 training samples.^40^ Each sampling method was tested across both XGBoost and Logistic Regression models and at every fine-tuning level, enabling comprehensive evaluation of model behavior under different retraining and resampling conditions.

### Model evaluation

We evaluated model performance following the original iPsRS study. Five predictive performance evaluation metrics were used: AUROC, precision, recall, F1 score, and specificity. Among these, AUROC served as the primary metric, measuring the model’s ability to distinguish between patients who experienced hospitalization within 1 year and those who did not.^41^^,^^42^ AUROC values were calculated for each model configuration using the held-out test set.

### Model explainability enhancement

To interpret model predictions, we used SHAP, a model-agnostic explainable AI technique.^24^ We generated SHAP summary plots for each model to assess the relative influence of individual-level SDoH variables on hospitalization risk. We also conducted causal structure learning using the mixed graphical model PC-stable (MGM-PC) algorithm, as described in the initial iPsRS study.^43–46^ This method constructs a causal graph based on conditional independence testing and was applied to the full external dataset. The resulting causal graphs were used to explore structural relationships among predictors and to identify potentially proximal causal risk factors for hospitalization.

### Fairness optimization

To assess model fairness, we used 7 group-level fairness metrics—including FNR, true positive rate, false positive rate, positive predictive value, negative predictive value, accuracy, and treatment equality (defined as FN/FP ratio).^47^ We considered FNR as the primary metric across 4 demographic subgroups: age (young vs old), sex (male vs female), and race/ethnicity (non-Hispanic Black [NHB] vs non-Hispanic White [NHW] and Hispanic vs non-Hispanic White). Age was dichotomized using a 65-year cutoff, with individuals younger than 65 classified as “young” and those 65 or older as “old.” For each subgroup comparison, FNR disparity was computed as the ratio of the protected group’s FNR to that of the privileged group. Ratios falling outside the 0.8-1.25 range were interpreted as indicative of fairness violations.^48^

Finally, to evaluate the threshold-finding component, we compared its results with Youden’s J statistic, defined as J=Sensitivity+Specificity-1.^49^ The threshold that maximized Youden’s J was selected, providing a direct optimization of the tradeoff between sensitivity and specificity.

## Results

The detailed summary of the cohort characteristics stratified by race/ethnicity is presented in Table 1. This study cohort included 17 857 adult patients from UF Health. The overall median age was 45.8 years, with notable age differences observed across racial and ethnic subgroups (eg, 47.9 for non-Hispanic White vs 42.4 for non-Hispanic Black, *P < .*001). The cohort was 70% female, with similar sex distributions across subgroups. Race-ethnicity groups were predominantly NHW (62.6%), followed by NHB (23.1%), Hispanic (7.7%), and other or unknown groups (6.6%). Educational attainment varied, with 29.7% of patients having college-level education or above, and 14.4% having high school or lower education. Most patients (93%) were flagged as experiencing financial constraints. Stable housing was documented for 52.4% of patients, while 10.9% were noted as homeless or living in a shelter. NLP-extracted SDoH, such as smoking, alcohol use, and drug use had high missingness, with >99% of records labeled as “unknown,” and insurance type was dominated by self-pay patients (NOPAY: 99.9%) as well.

**Table 1.** Characteristics of the study cohort (*N* = 17 857).

	Overall (*n* = 17 857)	NHW (*n* = 11 177)	NHB (*n* = 4 124)	Hispanic (*n* = 1 379)	Others (*n* = 1 177)	*P*-value
Age	45.76	47.96	42.4	41.55	41.59	<.001
Sex						0.1025
Female	12 491 (70.0%)	7496 (67.1%)	3273 (79.4%)	962 (69.8%)	760 (64.6%)	
Male	5366 (30.0%)	3681 (32.9%)	851 (20.6%)	417 (30.2%)	417 (35.4%)	
Race/Ethnicity						<.001
Non-Hispanic White	11 177 (62.6%)	11 177 (100.0%)	0 (0%)	0 (0%)	0 (0%)	
Non-Hispanic Black	4124 (23.1%)	0 (0%)	4124 (100.0%)	0 (0%)	0 (0%)	
Hispanic	1379 (7.7%)	0 (0%)	0 (0%)	1379 (100.0%)	0 (0%)	
Other	1142 (6.4%)	0 (0%)	0 (0%)	0 (0%)	1142 (97.0%)	
Unknown	35 (0.2%)	0 (0%)	0 (0%)	0 (0%)	35 (3.0%)	
Education						<.001
College or above	5309 (29.7%)	3233 (28.9%)	1343 (32.6%)	394 (28.6%)	339 (28.8%)	
High school or lower	2574 (14.4%)	1579 (14.1%)	714 (17.3%)	159 (11.5%)	122 (10.4%)	
Other	9974 (55.9%)	6365 (56.9%)	2067 (50.1%)	826 (59.9%)	716 (60.8%)	
Employment						<.001
Employed	156 (0.9%)	97 (0.9%)	33 (0.8%)	13 (0.9%)	13 (1.1%)	
Unemployed	236 (1.3%)	132 (1.2%)	74 (1.8%)	16 (1.2%)	14 (1.2%)	
Retired/disable	485 (2.7%)	358 (3.2%)	91 (2.2%)	18 (1.3%)	18 (1.5%)	
Other	16 980 (95.1%)	10 590 (94.7%)	3926 (95.2%)	1332 (96.6%)	1132 (96.2%)	
Financial						1
Has financial constraints	16 615 (93.0%)	10 418 (93.2%)	3853 (93.4%)	1261 (91.4%)	1083 (92.0%)	
Other	1242 (7.0%)	759 (6.8%)	271 (6.6%)	118 (8.6%)	94 (8.0%)	
Insurance						0.2503
Medicaid	1 (0.0%)	1 (0.0%)	0 (0%)	0 (0%)	0 (0%)	
Medicare	3 (0.0%)	3 (0.0%)	0 (0%)	0 (0%)	0 (0%)	
NOPAY	17 848 (99.9%)	11 170 (99.9%)	4122 (100.0%)	1379 (100.0%)	1177 (100.0%)	
Other	2 (0.0%)	2 (0.0%)	0 (0%)	0 (0%)	0 (0%)	
Private	2 (0.0%)	1 (0.0%)	1 (0.0%)	0 (0%)	0 (0%)	
Unknown	1 (0.0%)	0 (0%)	1 (0.0%)	0 (0%)	0 (0%)	
Housing stability						<.001
Homeless	1947 (10.9%)	963 (8.6%)	730 (17.7%)	145 (10.5%)	109 (9.3%)	
Stable	9363 (52.4%)	6092 (54.5%)	1793 (43.5%)	819 (59.4%)	659 (56.0%)	
Unknown	6547 (36.7%)	4122 (36.9%)	1601 (38.8%)	415 (30.1%)	409 (34.7%)	
Food insecurity						0.3413
Nourished/ well-develop	17 205 (96.3%)	10 749 (96.2%)	4010 (97.2%)	1321 (95.8%)	1125 (95.6%)	
Unknown	652 (3.7%)	428 (3.8%)	114 (2.8%)	58 (4.2%)	52 (4.4%)	
Marital status						<.001
Marital single	1278 (7.2%)	593 (5.3%)	460 (11.2%)	113 (8.2%)	112 (9.5%)	
Has partner	4125 (23.1%)	2900 (25.9%)	512 (12.4%)	394 (28.6%)	319 (27.1%)	
Widow_divorce	923 (5.2%)	614 (5.5%)	207 (5.0%)	66 (4.8%)	36 (3.1%)	
Other	11 531 (64.6%)	7070 (63.3%)	2945 (71.4%)	806 (58.4%)	710 (60.3%)	
Smoking status						0.1361
Yes	7 (0.0%)	5 (0.0%)	0 (0%)	2 (0.1%)	0 (0%)	
No	127 (0.7%)	92 (0.8%)	14 (0.3%)	8 (0.6%)	13 (1.1%)	
Unknown	17 723 (99.2%)	11 080 (99.1%)	4110 (99.7%)	1369 (99.3%)	1164 (98.9%)	
Alcohol use						0.139
Yes	7 (0.0%)	4 (0.0%)	0 (0%)	3 (0.2%)	0 (0%)	
No	126 (0.7%)	92 (0.8%)	14 (0.3%)	7 (0.5%)	13 (1.1%)	
Unknown	17 724 (99.3%)	11 081 (99.1%)	4110 (99.7%)	1369 (99.3%)	1164 (98.9%)	
Drug abuse						1
Yes	0 (0%)	0 (0%)	0 (0%)	0 (0%)	0 (0%)	
No	0 (0%)	0 (0%)	0 (0%)	0 (0%)	0 (0%)	
Unknown	17 857 (100.0%)	11 177 (100.0%)	4124 (100.0%)	1379 (100.0%)	1177 (100.0%)	

Values represent counts and percentages unless otherwise noted. P-values reflect unadjusted associations of each characteristic with the study outcome (all-cause hospitalization within 1 year), and are provided for descriptive purposes only. Chi-squared test was used for categorical variables and T-test was used for continuous variables. Both the statistical tests were 2-sided and no adjustments for multiple comparisons

We evaluated model performance across fine-tuning levels, as summarized in **Figure S2**. Without transferring adaptation, the original iPsRS model performed poorly on this external dataset (eg, AUROC = 0.398 for XGBoost with RUS and 0.410 with ROS/Normal). These below-chance results occurred only during baseline cross-cohort evaluation before local adaptation, likely reflecting a distribution shift between cohorts rather than the performance of the adapted models. With just 10% of local data used for fine-tuning, AUROC increased substantially and continued to improve with higher proportions. Among all configurations, the XGBoost model with Normal sampling and 70% fine-tuning achieved the best performance (AUROC = 0.671), followed closely by the same model with RUS (AUROC = 0.669) and ROS **(**AUROC = 0.665**)** sampling. Normal sampling shows a slight (but not statistically meaningful; <1% AUROC) advantage over ROS and RUS. This is likely because, in our dataset, ML models have sufficient signal to learn effectively under the original class distribution while preserving subgroup diversity and the natural distributional properties that support generalization. By contrast, resampling artificially alters the training distribution—ROS largely duplicates minority examples (adding little new information), and RUS discards majority examples (potentially removing informative negatives). As a result, these manipulations do not provide a clear benefit to predictive discrimination and can even mildly reduce performance. These findings indicate that resampling was useful as an experimental comparator for assessing class-imbalance strategies but did not provide a performance advantage in this cohort.

**Figure 2.**
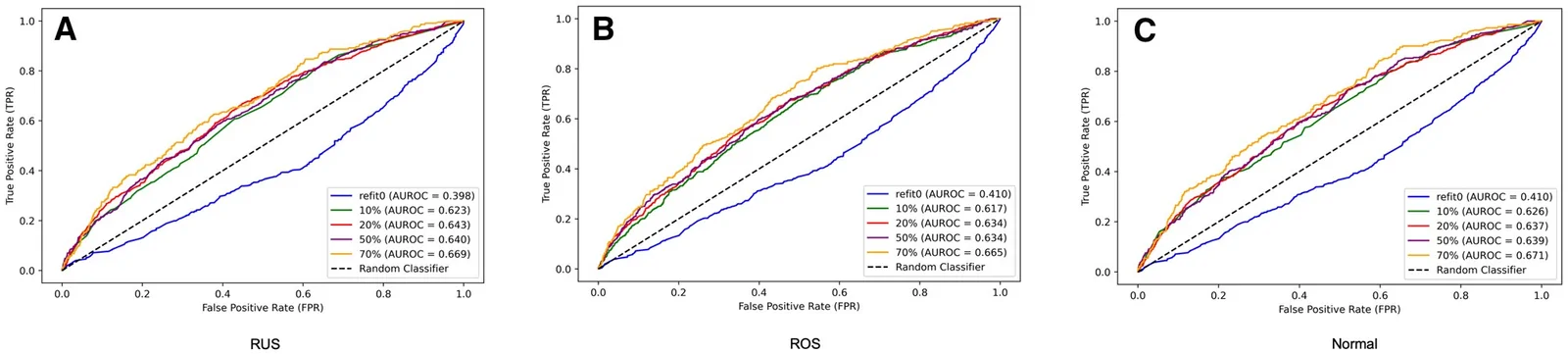
ROC curves of XGBoost models under 3 sampling strategies across 5 fine-tuning levels. (A) Performance across fine-tuning levels with RUS settings. (B) Performance across fine-tuning levels with ROS settings. (C) Performance across fine-tuning levels without resampling.

Logistic Regression models demonstrated a similar trend of improvement with increasing fine-tuning training samples, though overall performance remained lower than that of XGBoost. The best-performing Logistic Regression model, trained with Normal sampling and 70% fine-tuning, achieved an AUROC of 0.657. Models with RUS and ROS sampling underperformed slightly (maximum AUROC = 0.645) but still showed notable gains from the baseline (AUROC ≈ 0.445 at 0% fine-tuning). These results suggest that while logistic regression can benefit from local adaptation, XGBoost offer superior discrimination in this setting. ROC curves and the corresponding AUROC values for XGBoost models are shown in Figure 2. ROC curves for Logistic Regression models are included in **Figure S1**.

**Figure 3.**
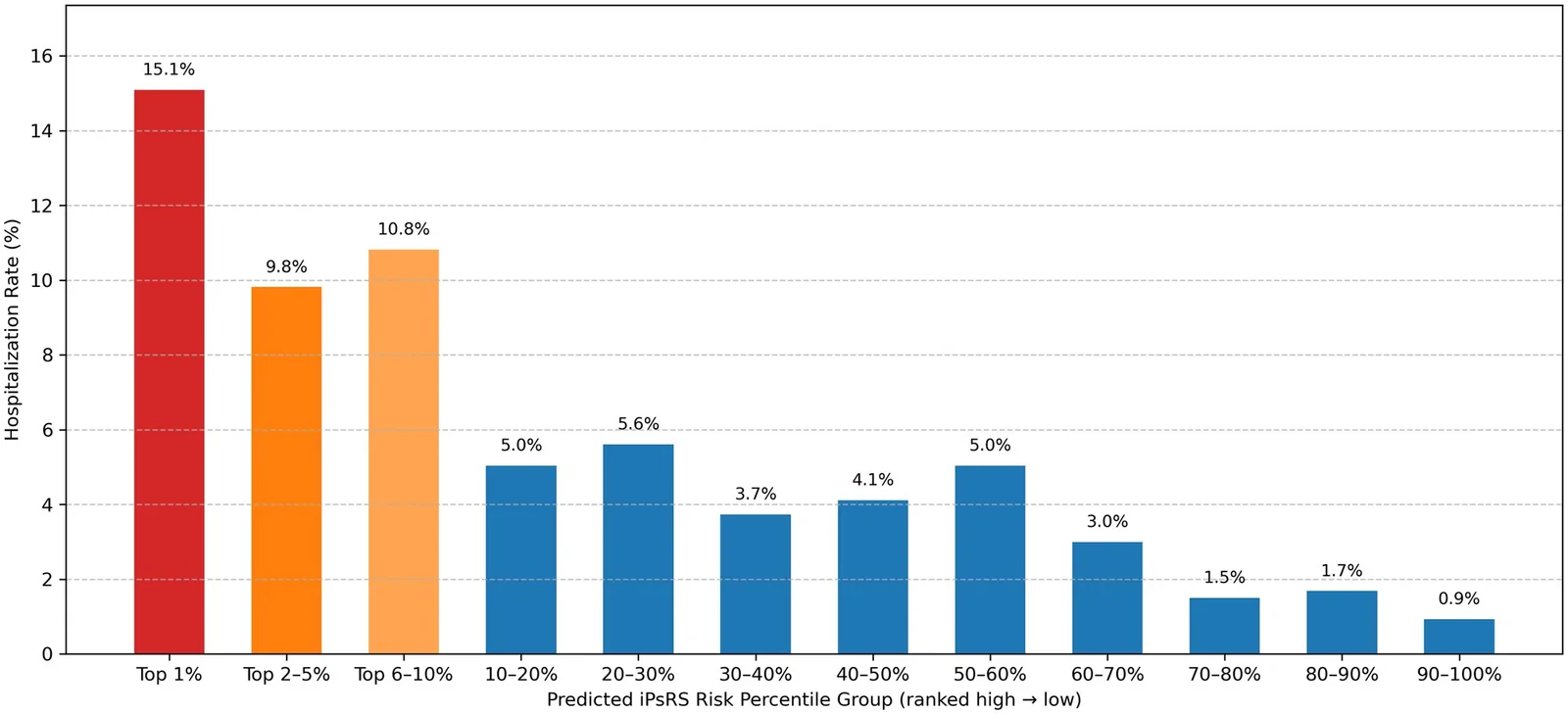
One-year hospitalization rates across iPsRS scores distribution. Rates are based on predictions from the XGBoost model trained with Normal sampling at 70% fine-tuning and calculated on the held out 30% test set. Bars are ordered from highest to lowest predicted risk, with colors denoting risk strata (top 1%, top 2%-5%, top 6%-10%, and remaining percentiles).

To evaluate risk stratification, patients were grouped into deciles based on iPsRS scores generated by the best-performing configuration (XGBoost with Normal sampling and 70% fine-tuning). The top decile was subdivided into top 1%, top 2%-5%, and top 6%-10% to provide finer differentiation among very high-risk patients. Prior work has shown that focusing on extreme-risk patients improves the clinical utility of risk stratification and helps reduce unnecessary alerts.^50^ As shown in Figure 3, the 1-year hospitalization rate increased from 0.9% (90%-100%) in the lowest decile to 15.1% in the highest (top 1%), demonstrating the model’s ability to meaningfully separate high- and low-risk individuals in the adaptation cohort.

**Figure 4.**
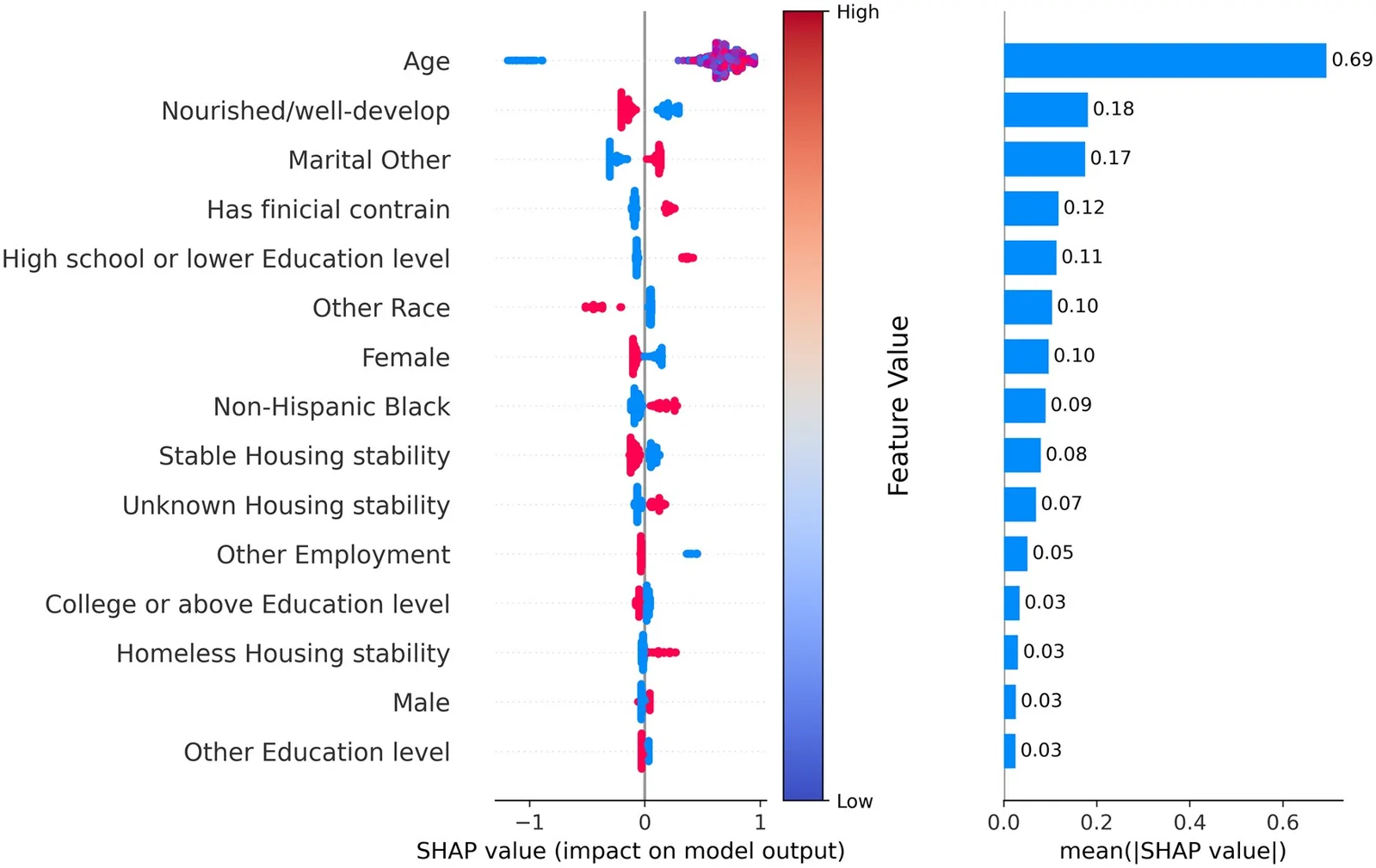
SHAP summary plot for the XGBoost model with Normal sampling and 70% fine-tuning. Features are ranked by mean absolute SHAP value.

Model interpretability was evaluated using SHAP values across all model configurations. As shown in Figure 4, the XGBoost model identifies age, food insecurity, marital status, and financial constraints as the most influential predictors of hospitalization. Importantly, SHAP analyses from the Logistic Regression model (**Figure S3**) also highlighted age as the top-ranking feature, followed by variables such as food insecurity, housing stability, and race. While the exact order of feature importance varied slightly across models and sampling strategies, both modeling approaches consistently emphasized key social risk domains such as housing, income, and education.

**Figure 5.**
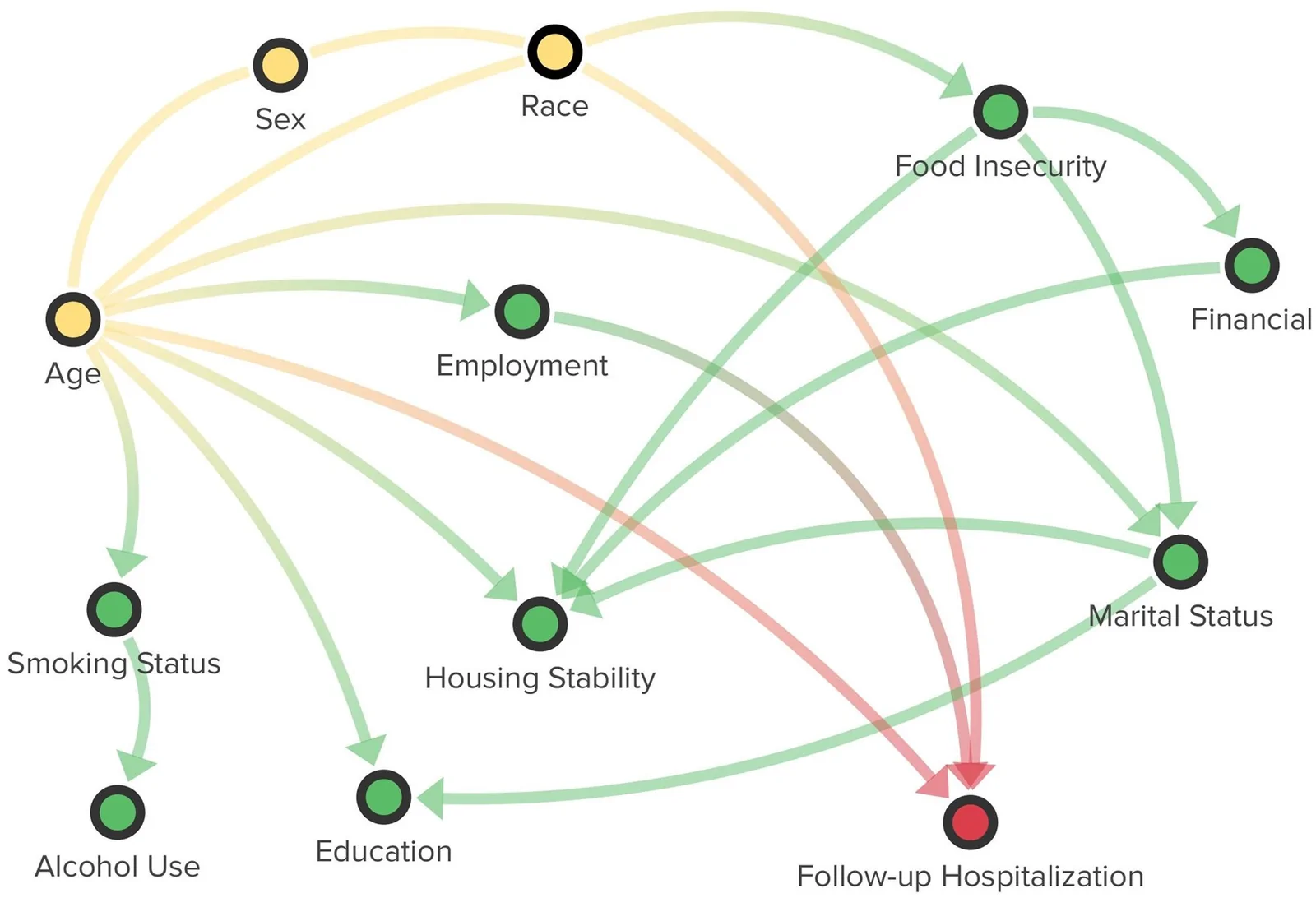
Maximally oriented partially directed acyclic graph estimated from the full external dataset using MGM-PC. Arrows represent conditional dependencies. The yellow nodes present demographics, green nodes stand for the individual-level SDoH, and the red node is the outcome (hospitalization).

We applied the MGM-PC algorithm to estimate a maximally oriented partially directed acyclic graph (MPDAG) for the study cohort in order to investigate the underlying causal structure among the SDoH and the outcome. The resulting MPDAG, shown in Figure 5, identifies age, race, and employment status as direct predictors of hospitalization. This result aligns closely with SHAP analysis and clinical intuition, as these factors are often independently associated with health deterioration and care needs.^51–53^ The causal graph further illustrates how age functions as a central upstream driver, with directed edges to variables such as employment, education, marital status, housing stability, and smoking status. Through these connections, age indirectly influences hospitalization via multiple social and behavioral pathways. Notably, drug abuse and insurance type were excluded from the final MPDAG due to near-constant values in the dataset. Specifically, drug abuse was recorded as 100% “unknown” and lacked any usable variability. Similarly, insurance type was labeled as “No Pay” in 99.9% of test cases, rendering it uninformative for causal discovery under the MGM-PC-Stable framework. Together, the causal graph and SHAP results offer complementary perspectives: while SHAP quantifies predictive contributions, the MPDAG reveals causal structure and pathways across the SDoH landscape. Both reinforce the importance of age, race, and employment status as key levers of health risk and equity, and highlight the interconnected social mechanisms that shape hospitalization outcomes.

We assessed fairness by examining FNR disparities across 3 demographic dimensions: age (older adults ≥ 65 vs younger adults), sex (male vs female), and race/ethnicity (non-Hispanic Black vs non-Hispanic White and Hispanic vs non-Hispanic White) on XGBoost model (Table 2). Age-based disparities were among the most pronounced, especially under RUS and ROS. The model trained with RUS showed a substantial imbalance, with an FNR disparity of 2.078 for young vs old patients, suggesting that younger individuals were significantly more likely to be misclassified as low risk. This disparity decreased under ROS (1.518) and was minimal under Normal sampling (1.003), highlighting the sensitivity of age-related predictions to resampling strategies. For sex, the RUS-trained model yielded an FNR disparity of 1.303 for male vs female patients, indicating potential bias against males. This disparity reduced to 1.108 under ROS and was nearly eliminated under Normal sampling (1.004), suggesting better sex fairness under the baseline setting.

**Table 2.** Summary of FNRs and FNR disparity ratios across all subgroups and sampling methods (protected vs privileged defined as: young vs old, male vs female, non-Hispanic Black vs non-Hispanic White, and Hispanic vs non-Hispanic White).

Sampling	Subgroup	Protected FNR	Privileged FNR	FNR disparity
Normal	Young vs old	0.999	0.996	1.003
RUS	Young vs old	0.482	0.232	2.078
ROS	Young vs old	0.642	0.423	1.518
Normal	Male vs female	0.999	0.995	1.004
RUS	Male vs female	0.431	0.331	1.303
ROS	Male vs female	0.587	0.530	1.108
Normal	NHB vs NHW	0.998	0.998	1.000
RUS	NHB vs NHW	0.375	0.388	0.967
ROS	NHB vs NHW	0.581	0.531	1.096
Normal	Hispanic vs NHW	0.989	0.998	0.991
RUS	Hispanic vs NHW	0.536	0.388	1.366
ROS	Hispanic vs NHW	0.771	0.531	1.455

Regarding racial and ethnic subgroups, the disparity between non-Hispanic Black and non-Hispanic White patients remained close to parity across all sampling strategies (1.0 under Normal, 0.967 under RUS, and 1.096 under ROS). However, the Hispanic vs non-Hispanic White comparison showed more variation. Under RUS, the disparity rose to 1.366, and it further increased to 1.455 under ROS, while it stayed within acceptable range under Normal sampling (0.991). The full set of fairness metrics is summarized in **Table S3**.

### Threshold-finding experiment

To select an operating threshold for the threshold-finding experiment (XGBoost with Normal sampling at 70% fine-tuning), we compared Youden’s J statistic with a knee-guided multi-objective (MOO) procedure, evaluated without and with normalization. As shown in Table 3, the maximum Youden’s J was 0.241 at a threshold of 0.36, which favored sensitivity (recall 0.896) but yielded low precision (0.056), low accuracy (0.368), a modest F1 score (0.105), and a large positive cohort size (*n* = 3,559). The non-normalized MOO knee identified a threshold of 0.87, producing very high specificity (0.999) and accuracy (0.958) but near-zero recall (0.009), a very small positive cohort (*n* = 5), and a low F1 score (0.018). The normalized MOO knee selected a threshold of 0.69, providing a more balanced profile (accuracy 0.860, precision 0.106, recall 0.320, specificity 0.883, F1 0.159; cohort size *n* = 671). We therefore used the normalized knee threshold of 0.69 for subsequent threshold-dependent analyses because it avoids the extremes of over-calling and under-calling positives while maintaining a better balance between precision and recall.

**Table 3.** Comparison of threshold selection methods on the evaluation split (XGBoost with normal sampling at 70% fine-tuning).

Method	Threshold	Accuracy	Precision	Recall	Specificity	F1	Cohort size
Youden’s J (max J)	0.36	0.368	0.056	0.896	0.345	0.105	3559
Knee-point (MOO, non-normalized)	0.87	0.958	0.400	0.009	0.999	0.018	5
Knee-point (MOO, normalized)	0.69	0.860	0.106	0.320	0.883	0.159	671

Details are provided in the **Supplementary Materials**. **Figure S4** shows parallel coordinates plots of the Pareto front (normalized and non-normalized). **Figure S5** shows confusion matrices at thresholds 0.36, 0.69, and 0.87, with the selected threshold of 0.69 highlighted. Complete threshold-sweeping performance metrics are reported in **Table S2**.

## Discussion

This study demonstrates that the iPsRS framework, originally developed using a disease-specific population of individuals with T2D, generalizes effectively to a broader adult patient cohort. After fine-tuning with local data, the adapted models exhibited moderate predictive performance, achieving AUROC 0.671 comparable to those reported in the original study. Notably, the observed level of predictive performance should be interpreted in the context of real-world clinical prediction. In clinical prediction modeling, AUROC values in the range of approximately 0.6-0.7 are commonly interpreted as reflecting moderate discriminative ability and can still provide meaningful risk stratification for population-level screening and decision support. Recent evidence also suggests that ML models with similar predictive performance may still offer practical value in real-world clinical settings.^54^^,^^55^ Importantly, our model relies exclusively on individual-level SDoH variables without incorporating clinical predictors. Prior studies suggest that SDoH can complement clinical and EHR-derived predictors, although the magnitude of improvement varies across populations, outcomes, and data sources.^56–58^ Studies using SDoH information have also reported limited or inconsistent improvements in overall discrimination, particularly when SDoH variables are used without detailed clinical information or are represented using community-level measures.^55^^,^^58^ This context helps explain the moderate AUROC observed in our SDoH-only hospitalization model. Nevertheless, SDoH-informed risk screening may still support population-level risk stratification by helping identify patients who may benefit from social support, community-resource referral, and care coordination.^59^ Beyond predictive accuracy, iPsRS also shows potential for real-world clinical use. Instead of collecting SDoH data without clear follow-up, iPsRS supports clinicians by automatically screening patients for elevated hospitalization risk linked to social factors, helping clinicians direct care to those who need it. To support real-world adoption, our proposed iPsRS has been prototyped for integration into the Epic EHR system using a user-centered design approach guided by the Five Rights of Clinical Decision Support framework.^60^^,^^61^ Within Epic, hospitalization risk scores appear in physician workflows through Best Practice Advisories and Navigator modules, while case managers and social workers can access more detailed information via an interactive web-based interface. This integration ensures risk information reaches the right user at the right time, enabling timely referrals to social work and community resources, and translating SDoH screening into actionable interventions within routine care.

To further understand how the model makes predictions, we examined its interpretability using SHAP and causal structure learning. Beyond predictive modeling, SHAP analysis shows age and food insecurity consistently emerged as the most influential predictors. Age likely captures baseline morbidity and physiological reserve, whereas food insecurity—an indicator of material deprivation and reduced physiologic reserve—heightens vulnerability to hospitalization. In addition, the use of causal structure learning provided insight into upstream mechanisms driving hospitalization risk. The learned causal graph identified age, race, and employment as direct precursors of hospitalization. These findings complement the SHAP-based feature attribution by highlighting not only which features are predictive but also how they may causally interact within a broader social context. To compare causal relationships with model-learned feature importance, we analyzed SHAP values from the XGBoost model trained with Normal sampling and fine-tuned on 70% of the general adult dataset. SHAP ranking (Figure 4) confirmed age as the dominant predictor and placed race indicators mid-to-high, consistent with their proximity in the causal graph. However, other highly ranked SHAP features, such as food insecurity and financial constraints, were not connected to hospitalization in the causal graph. By contrast, employment, although a direct parent of hospitalization, showed a smaller SHAP contribution. These discrepancies reflect the fundamental difference between predictive importance and structural causality. SHAP quantifies how much each feature contributes to prediction given the available data, regardless of underlying causal direction. In contrast, the causal graph reveals potential pathways of influence but is sensitive to variable distributions, data quality, and assumptions about independence.

Finally, to evaluate the equity of model predictions, we examined fairness metrics across demographic groups. Our fairness analysis in Table 2 indicates that resampling choices change equity tradeoffs. RUS produced the largest FNR disparities, especially for age and for the Hispanic vs non-Hispanic White comparison. ROS reduced some gaps but did not remove them. Notably, the Normal sampling configuration showed both the highest AUROC and the most balanced FNR distribution across demographic groups, indicating that moderate predictive performance and subgroup equity can be achieved simultaneously.

### Limitations

Substantial missingness in individual-level SDoH variables, particularly for smoking, alcohol use, and drug use, was handled by explicitly modeling an “unknown” category rather than 0 imputation. While this approach reflects real-world EHR documentation practices, it limits the direct interpretability of some social risk factors. In addition, the dominance of self-pay insurance in the cohort constrained the variability and potential contribution of insurance-related features. Social determinants of health variables derived from keyword-based NLP are also subject to misclassification and extraction bias due to heterogeneity in clinical documentation.

The original iPsRS was developed in a T2D-specific population, and its application to a broader adult primary care cohort required fine-tuning, underscoring potential limitations in external validity. The modest cohort size further raises the possibility of sampling bias and may limit the stability of subgroup-specific performance and fairness estimates. In addition, the low hospitalization prevalence introduced class imbalance, which contributed to modest F1 scores and limited the interpretability of threshold-dependent metrics. Although resampling methods were explored, they did not meaningfully improve overall performance. Our evaluation primarily emphasized AUROC and threshold-dependent metrics, and we did not systematically evaluate precision-recall performance or calibrate models using AUPRC-oriented objectives. Because AUPRC is more informative than AUROC under substantial class imbalance, future work should examine precision-recall calibration, AUPRC-based model selection, and threshold optimization to better identify high-risk patients. Fairness assessment in this study focused primarily on FNR disparities; other formal fairness definitions, such as equalized odds or disparate impact, were not systematically evaluated and warrant future investigation. While XGBoost showed modest performance gains, logistic regression offers advantages in simplicity and ease of clinical implementation, reflecting a tradeoff between performance and deployability.

Interpretability was assessed using SHAP, which provides insight into predictive contributions but does not support causal inference and may be influenced by correlated features. Complementary approaches, such as generalized linear models with interpretable effect estimates, may further enhance interpretability. Finally, the study period spanned January 2018 through June 2022, with a 1-year follow-up window, overlapping substantially with the COVID-19 pandemic. Changes in healthcare utilization and hospitalization patterns during this period may have influenced observed outcome rates and should be considered when interpreting model performance.

## Conclusion

This study demonstrates that the iPsRS framework, originally developed in a disease-specific context, can be effectively adapted to a general adult population using only individual-level SDoH. The adapted models maintained fair predictive performance, yielded clinically meaningful risk stratification, and uncovered fairness tradeoffs across demographic subgroups. Incorporating SDoH into risk prediction remains essential for promoting equitable and data-informed healthcare. Future extensions of this framework may further support population health management and the design of targeted interventions to mitigate avoidable hospitalizations and reduce social disparities in health outcomes.

## Supplementary Material

ooag161_Supplementary_Data

## Data Availability

The data presented in this study are available on request from the corresponding author. The data are not publicly available due to privacy restrictions.

## Code availability

The codes presented in this study are available on request from the corresponding author.
