# Supplementary material for: Adaptation of a fair individualized polysocial risk score for hospitalization risk prediction
Source: JAMIA Open. 2026 Aug 24;9(4):ooag161. doi: 10.1093/jamiaopen/ooag161 (PMC13503007; doi:10.1093/jamiaopen/ooag161)
Supplement: Supplementary file 1 [file ooag161_supplementary_data.zip › Supplemental file revision_20260716(clean).docx]

**Supplement Material**

**S1. Fairness Metrics Definition**

To evaluate fairness in the externally validated iPsRS model, we used a set of commonly accepted group fairness metrics. These include predictive parity, predictive equality, equalized odds, conditional use accuracy equality, treatment equality, equality of opportunity, and overall accuracy equality. Each of these measures examines the model’s behavior across different demographic groups (e.g., race, sex, age), defined as either protected or privileged.

• Predictive parity ensures that the positive predictive value (PPV) is similar for both protected and privileged groups.

• Predictive equality measures the false positive rate (FPR) across groups.

• Equalized odds requires both true positive rates (TPR) and FPR to be equal.

• Conditional use accuracy equality combines parity in both PPV and negative predictive value (NPV).

• Treatment equality assesses the ratio of false negatives (FN) to false positives (FP).

• Equality of opportunity evaluates parity in false negative rates (FNR).

• Overall accuracy equality considers balanced accuracy across subgroups.

**S2. Individual-Level SDoH Features**

Our model was trained and validated using structured, individual-level social determinants of health (SDoH) features. These include demographics (age, sex, race/ethnicity), socioeconomic factors (insurance, employment, education, housing), and behavioral health indicators (smoking, alcohol use, drug use). All features were derived from EHR-structured fields and a rule-based NLP pipeline for unstructured notes, with missing values was cleaned.

**Supplementary Table S1**. Variable Definitions and Value Labels

| **Variable Name** | **Type** | **Description** | **Value Labels** |
| --- | --- | --- | --- |
| age | Num | Patient age (in years) | Continuous |
| SEX_CD | Char | Patient sex | 0 = Female; 1 = Male |
| race_ethnicity | Char | Race and ethnicity | 0 = Non-Hispanic White (NHW); 1 = Non-Hispanic Black (NHB); 2 = Hispanic; 3 = Other; 4 = Unknown |
| insurance_type | Char | Primary insurance type | 0 = Private; 1 = Medicaid; 2 = Medicare; 3 = Other; 4 = Self-pay (NoPay); 5 = Unknown |
| EDUCATION_SDoH | Num | Educational attainment | 1 = College or above; 2 = High school or lower; 3 = Other |
| EMPLOYMENT_SDoH | Num | Employment status | 1 = Employed; 2 = Unemployed; 3 = Retired/Disabled; 4 = Other |
| FINANCIAL_SDoH | Num | Financial constraints | 1 = Has financial constraint; 2 = No constraint; 3 = Other |
| LIVING_CON_SDoH | Num | Housing stability | 1 = Homeless/Shelter; 2 = Stable housing; 3 = Other |
| LIVING_SUPLY_SDoH | Num | Food insecurity | 1 = Nourished/Well-developed; 2 = Poorly nourished/Poorly developed; 3 = Missing |
| MARITSTATUS_SDoH | Num | Marital status | 1 = Single; 2 = Married/Has partner; 3 = Widowed/Divorced; 4 = Other |
| SMOKING_SDoH | Num | Smoking status | 1 = Yes; 2 = No; 3 = Other |
| ALCOHOL_SDoH | Num | Alcohol use | 1 = Yes; 2 = No; 3 = Other |
| DRUGUSE_SDoH | Num | Drug use | 1 = Yes; 2 = No; 3 = Other |

Description and coding schema for the 13 individual-level social determinants of health variables used in the study, including age, sex, race/ethnicity, insurance status, education, employment, financial constraints, housing stability, food insecurity, marital status, smoking, alcohol, and drug use. Employment status was the only SDoH variable supplemented using information extracted from unstructured clinical notes. The following keywords were used to identify employment-related notes for subsequent rule-based processing.

**Keywords for filtering notes with employment status**

'employment', 'unemployment', 'social worker', 'homeless' 'worked in', 'work as', 'works as', 'working in', 'working at', 'working as', 'employed as', 'employed in', 'retired',

**S3. Model Adaptation**

We tested the transportability of the iPsRS model by progressively fine-tuning it on increasing proportions of a general patient dataset. Fine-tuning levels included 0%, 10%, 20%, 50%, and 70%, simulating deployment in varying new environments. Sampling strategies (normal, ROS, RUS) were combined with both logistic regression and XGBoost to assess performance robustness.

**Supplementary Figure S1**. ROC curves of Logistic Regression models under three sampling strategies across five fine-tuning levels.

This figure visualizes the discrimination performance of Logistic Regression across three sampling approaches: Random Undersampling (RUS), Random Oversampling (ROS), and Normal (no sampling). Each subpanel shows ROC curves corresponding to fine-tuning levels of 0%, 10%, 20%, 50%, and 70%. As seen in the figure, performance improves notably with even 10% of local data retraining. Without any adaptation (Refit 0%), AUROC values are consistently low (≈ 0.44), while models fine-tuned with 70% data achieve peak AUROC of 0.657 with Normal sampling. These results are consistent with the trend observed in the main text, where Logistic Regression models benefit from adaptation, though not to the extent of XGBoost.

A. XGBoost with Normal Sampling

B. XGBoost with Oversampling (ROS)

C. XGBoost with Undersampling (RUS)

F. Logistic Regression with Undersampling (RUS)

E. Logistic Regression with Oversampling (ROS)

D. Logistic Regression with Normal Sampling

**Supplementary Figure S2.** Model performance across fine-tune levels: XGBoost and Logistic Regression with varying sampling strategies.

Among the five evaluation metrics plotted—AUROC, Precision, Recall, F1 Score, and Specificity—AUROC provides the clearest indication of overall discrimination capability. It consistently improves with higher fine tune percentages across all settings, validating the benefit of local adaptation. Recall increases steadily with retraining, indicating better sensitivity to hospitalization outcomes. F1 Score also rises accordingly but remains low overall due to class imbalance, emphasizing the difficulty of achieving high precision in rare-event prediction. Precision remains relatively flat and low, while Specificity starts high (particularly at 0% fine tune) but slightly decreases as models adapt—reflecting the tradeoff between detecting positives and avoiding false alarms. These results suggest that fine tuning improves true positive identification at some cost to true negatives, which may be acceptable in high-stakes healthcare settings focused on minimizing missed risk.

**S4. Feature Contribution Analysis via SHAP**

To interpret model predictions and assess the contribution of each individual-level SDoH feature, we computed SHAP (SHapley Additive exPlanations) values for all trained models. SHAP provides a unified measure of feature importance by estimating each variable's marginal contribution to the model’s output, allowing for transparent interpretation of both linear and nonlinear models.

**Supplementary Figure S3.** SHAP summary plot for the Logistic Regression model with normal sampling and 70% fine-tuning. Features are ranked by mean absolute SHAP value.

This figure shows the SHAP summary plot for the Logistic Regression model trained with Normal Sampling and 70% fine-tuning. Unlike the XGBoost model—which emphasized a broader set of features including financial constraint and marital status—Logistic Regression places overwhelming weight on age, with all other features contributing marginally. This reflects the model’s limited capacity to capture complex interactions between SDoH variables. A few categorical SDoH indicators, such as financial constrain, employment status, housing stability, and race/ethnicity (e.g., NHB), appear with non-zero but relatively small SHAP values. The stark contrast with XGBoost highlights the advantages of tree-based methods in modeling nuanced, nonlinear associations among social risk factors and hospitalization risk.

**S5. Threshold-Finding Experiment**

**Supplementary Table S2** threshold_metrics_normal.csv — Threshold-sweeping performance metrics (accuracy, precision, recall, specificity, F1 score, AUROC, predicted-positive count) for the selected configuration, with Pareto-optimal thresholds flagged. These data correspond to the knee-guided multi-objective procedure described in the Methods and Results. Please see the table at the end of the material.

(A) Non-normalized Parallel coordinates plot

(B) Normalized Parallel coordinates plot

**Supplementary Figure S5.** Parallel coordinates plots of Pareto-optimal thresholds (front 0) for the threshold-finding experiment. **(A)** Non-normalized metrics. **(B)** Normalized metrics. Each vertical axis corresponds to one metric (accuracy, precision, recall, specificity, F1), and each green polyline represents a candidate threshold on the Pareto front. The red polyline marks the selected knee solution **(A: 0.87; B: 0.69).**

**Supplementary Figure S4.** Confusion matrices of the XGBoost model (normal sampling, 70% fine-tuning) at three thresholds selected by different methods: **0.36 (Youden’s J), 0.69 (knee-point, normalized MOO), and 0.87 (knee-point, non-normalized MOO).**

**S6. Fairness Evaluation by Subgroup**

We analyzed fairness metrics across four protected groups: race (NHB vs. NHW), ethnicity (Hispanic vs. NHW), sex (Male vs. Female), and age (Older adults ≥ 65 vs. younger adults). Metrics included PPV, NPV, FPR, FNR, TPR, treatment equality, and overall accuracy.

This table summarizes group fairness metrics for XGBoost models evaluated under three sampling methods: Normal Sampling, Random Oversampling (ROS), and Random Undersampling (RUS), across four protected subgroups: race (NHB vs. NHW), ethnicity (Hispanic vs. NHW), sex (Male vs. Female), and age (Older vs. Younger adults). For each group, we report Positive Predictive Value (PPV), False Positive Rate (FPR), True Positive Rate (TPR), Negative Predictive Value (NPV), False Negative Rate (FNR), Accuracy (ACC), and Treatment Equality (TE, defined as FN/FP). Under Normal sampling, models exhibited minimal subgroup disparities largely because sensitivity was uniformly low, reflected in extremely low TPR and near-perfect FNRs for protected groups (e.g., FNR ≈ 0.9985 for NHB patients; 0.9996 for younger adults). With ROS, subgroup recall improved markedly, particularly for race and ethnicity, although this gain came at the cost of substantially higher FPR disparities in age- and ethnicity-based comparisons. RUS further improved alignment of TPR and FNR across some groups—for example, NHB vs. NHW TPRs (0.6252 vs. 0.6123) and FNRs (~0.37–0.39)—but also introduced new imbalances, particularly in age and Hispanic vs. NHW comparisons, leading to a less stable fairness profile overall.

| Sampling | Group | protected_PPV | privileged_PPV | protected_FPR | privileged_FPR | protected_TPR | privileged_TPR | protected_NPV | privileged_NPV | protected_te | privileged_te | protected_FNR | privileged_FNR | protected_ACC | privileged_ACC |
| --- | --- | --- | --- | --- | --- | --- | --- | --- | --- | --- | --- | --- | --- | --- | --- |
| Normal | NHB vs. NHW | 0.1471 | 0.1462 | 0.0007 | 0.0004 | 0.0015 | 0.0016 | 0.9479 | 0.9585 | 9.0950 | 27.3598 | 0.9985 | 0.9984 | 0.9472 | 0.9581 |
| Normal | Young vs. Old | 0.0898 | 0.1894 | 0.0003 | 0.0014 | 0.0008 | 0.0045 | 0.9642 | 0.9382 | 11.5437 | 15.0868 | 0.9992 | 0.9955 | 0.9639 | 0.9370 |
| Normal | Male vs. Female | 0.1187 | 0.2171 | 0.0004 | 0.0010 | 0.0009 | 0.0047 | 0.9601 | 0.9543 | 18.832 | 10.6627 | 0.9991 | 0.9953 | 0.9597 | 0.9534 |
| Normal | Hispanic vs. NHW | 0.3551 | 0.1462 | 0.0006 | 0.0004 | 0.0109 | 0.0016 | 0.9734 | 0.9585 | 0.7475 | 27.3598 | 0.9891 | 0.9984 | 0.9728 | 0.9581 |
| ROS | Hispanic vs. NHW | 0.0585 | 0.0665 | 0.1012 | 0.2873 | 0.2288 | 0.4695 | 0.9766 | 0.9689 | 0.2870 | 0.0834 | 0.7712 | 0.5305 | 0.8803 | 0.7026 |
| ROS | Young vs. Old | 0.0561 | 0.0843 | 0.2240 | 0.4160 | 0.3585 | 0.5764 | 0.9702 | 0.9543 | 0.1101 | 0.0719 | 0.6415 | 0.4236 | 0.7610 | 0.5833 |
| ROS | NHB vs. NHW | 0.0688 | 0.0665 | 0.3130 | 0.2873 | 0.4189 | 0.4695 | 0.9556 | 0.9689 | 0.1053 | 0.0834 | 0.5811 | 0.5305 | 0.6729 | 0.7026 |
| ROS | Male vs. Female | 0.0607 | 0.0783 | 0.2662 | 0.2671 | 0.4126 | 0.4699 | 0.9678 | 0.9665 | 0.0953 | 0.1005 | 0.5874 | 0.5301 | 0.7209 | 0.7208 |
| RUS | NHB vs. NHW | 0.0631 | 0.0593 | 0.5117 | 0.4219 | 0.6252 | 0.6123 | 0.9596 | 0.9718 | 0.0417 | 0.0404 | 0.3748 | 0.3877 | 0.4954 | 0.5795 |
| RUS | Male vs. Female | 0.0538 | 0.0687 | 0.4166 | 0.4370 | 0.5689 | 0.6691 | 0.9702 | 0.9726 | 0.0437 | 0.0375 | 0.4311 | 0.3309 | 0.5828 | 0.5678 |
| RUS | Hispanic vs. NHW | 0.0420 | 0.0593 | 0.3021 | 0.4219 | 0.4703 | 0.6123 | 0.9793 | 0.9718 | 0.0536 | 0.0404 | 0.5297 | 0.3877 | 0.6916 | 0.5795 |
| RUS | Young vs. Old | 0.0492 | 0.0778 | 0.3719 | 0.6020 | 0.5178 | 0.7681 | 0.9723 | 0.9632 | 0.0487 | 0.0264 | 0.4822 | 0.2319 | 0.6241 | 0.4208 |
|  |  |  |  |  |  |  |  |  |  |  |  |  |  |  |  |

**Supplementary Table S3. Fairness metrics for XGBoost models across sampling strategies and subgroups** (protected vs. privileged defined as: Young vs. Old, Male vs. Female, NHB vs. NHW, and Hispanic vs. NHW)
